# Observations of discrete harmonics emerging from equatorial noise

**DOI:** 10.1038/ncomms8703

**Published:** 2015-07-14

**Authors:** Michael A. Balikhin, Yuri Y. Shprits, Simon N. Walker, Lunjin Chen, Nicole Cornilleau-Wehrlin, Iannis Dandouras, Ondrej Santolik, Christopher Carr, Keith H. Yearby, Benjamin Weiss

**Affiliations:** 1 Department of Automatic Control and Systems Engineering, University of Sheffield, Mappin Street, Sheffield S1 3JD, UK; 2 Department of Earth Planetary and Space Sciences, UCLA, 595 Charles Young Drive East, Box 951567, Los Angeles, California 90095-1567, USA; 3 Department of Earth Atmospheric and Planetary Sciences, MIT, 77 Massachusetts Avenue, Cambridge, Massachusetts 02139-4307, USA; 4 W.B. Hanson Center for Space Sciences, Department of Physics, The University of Texas at Dallas, 800 West Campbell Road, Richardson, Texas 75080-3021, USA; 5 LPP, CNRS, École Polytechnique, Palaiseau 91128, France; 6 LESIA, Observatoire de Paris, Section de Meudon, 5, Place Jules Janssen, Meudon 92195, France; 7 CNRS, IRAP, 9, Avenue du Colonel Roche, Toulouse BP 44346-31028, France; 8 UPS-OMP, IRAP, 14, Avenue Edouard Belin, Toulouse 31400, France; 9 Department of Space Physics, Institute of Atmospheric Physics ASCR, Bocni II/1401, 14131 Praha 4, Czech Republic; 10 Faculty of Mathematics and Physics, Charles University in Prague, V Holesovickach 2, 18000 Praha 8, Czech Republic; 11 Blackett Laboratory, Imperial College London, South Kensington Campus, London SW7 2AZ, UK

## Abstract

A number of modes of oscillations of particles and fields can exist in space plasmas. Since the early 1970s, space missions have observed noise-like plasma waves near the geomagnetic equator known as ‘equatorial noise'. Several theories were suggested, but clear observational evidence supported by realistic modelling has not been provided. Here we report on observations by the Cluster mission that clearly show the highly structured and periodic pattern of these waves. Very narrow-banded emissions at frequencies corresponding to exact multiples of the proton gyrofrequency (frequency of gyration around the field line) from the 17th up to the 30th harmonic are observed, indicating that these waves are generated by the proton distributions. Simultaneously with these coherent periodic structures in waves, the Cluster spacecraft observes ‘ring' distributions of protons in velocity space that provide the free energy for the waves. Calculated wave growth based on ion distributions shows a very similar pattern to the observations.

## Abstract (web summary)

Since the 1970s space missions have observed `equatorial noise' — noise-like plasma waves closely confined to the magnetic equatorial region of Earth s magnetosphere. Here, the authors uncover their structured and periodic frequency pattern, revealing that they are generated by proton distributions.

## Graphical abstract

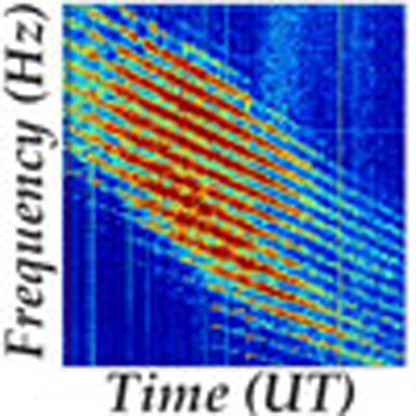

Oscillations of the electric and magnetic field in plasmas, usually referred to as plasma waves, have been observed in the Earth's magnetosphere, interplanetary space, and most recently, outside of the heliosphere. In space, plasma waves exhibit a wide variety of modes and are classified according to their frequency, polarization characteristics, types of oscillation (longitudinal or transverse) and their dispersion relation, which is the relation between the frequency of the wave and its vector of propagation.

The OGO (Orbiting Geophysical Observatory) 3 space mission detected plasma waves that were very closely confined to the terrestrial magnetic equatorial region^1^,^2^. These emissions were observed above the proton gyrofrequency—the frequency at which a proton gyrates around the field line. Due to their close confinement to the equator, they were named ‘equatorial noise', but are also referred to as fast magnetosonic waves or magnetosonic noise due to their properties. These emissions are one of the most common waves observed in space. While these waves are observed only very close to the geomagnetic equator, they are seen on around 60% of equatorial satellite traversals in the inner magnetosphere^3^. When these waves were discovered^1^, it was also noted that they may also be in resonance with harmonics of electron bounce motion (periodic motion of trapped electrons along the field line between the mirror points) and thus may be potentially generated by electrons in the plasma.

Observations by Interplanetary Monitoring Platform 6 Satellite, Hawkeye 1 (Explorer 52) Satellite and the Geostationary Operational Environmental Satellite^2^,^4^ showed cursory evidence for discrete frequency bands, suggesting that these waves may interact with protons, alpha particles and heavy ions trapped near the equator. However, the width and spacing of these bands in frequency appeared to be non-uniform and could not be accurately measured, except at the frequencies of the lowest harmonics. The spectral frequencies of these bands were in some cases approximately at harmonics of the proton gyrofrequency but did not match them exactly (see Supplementary Note 2). Clear observational and analytical evidence for this type of frequency spectrum has so far remained elusive. A number of very detailed follow-up studies, including a recent detailed statistical study using measurements from the Polar mission^5^, either failed to find the discrete waves, or found spectral structures at frequencies different from harmonics of the local proton gyrofrequency^6^. The suggested explanation for the discrepancy between theory and observations was that the waves may be generated at different locations (near the equator) and propagate to the point of observation. However, since observations showing a clear harmonic structure were not available, the theory remained unverified by observations.

Multi-point Cluster observations presented in this study show remarkable observations of very distinct harmonic emissions coinciding with multiples of gyrofrequency on two Cluster spacecraft. The waves are observed exactly in the source region. Using the observed distributions of rings, we calculated the growth rates of magnetosonic waves and show that the results of the calculations are consistent with the observed harmonics between the 17th and 30th harmonic resonances. The presented observations of distinct periodic emissions exactly at the harmonics of the gyrofrequency together with the simulations of wave growth that are based on the observed ion distributions, definitively show that magnetosonic emissions are generated by unstable ion ring distributions.

## Results

### Cluster mission

The European Space Agency (ESA) Cluster mission^7^ consists of four identically instrumented spacecraft in a polar, eccentric orbit (apogee 18.6, perigee 3 Earth radii) with a period of 57 h. Launched in August 2000, the mission has been operating since February 2001. During its lifetime, the inter-satellite separation has varied from less than a few hundred kilometres to over 20,000 km, to explore processes occurring within the magnetosphere at different spatial scales (see Supplementary note 3).

**Figure 1:**
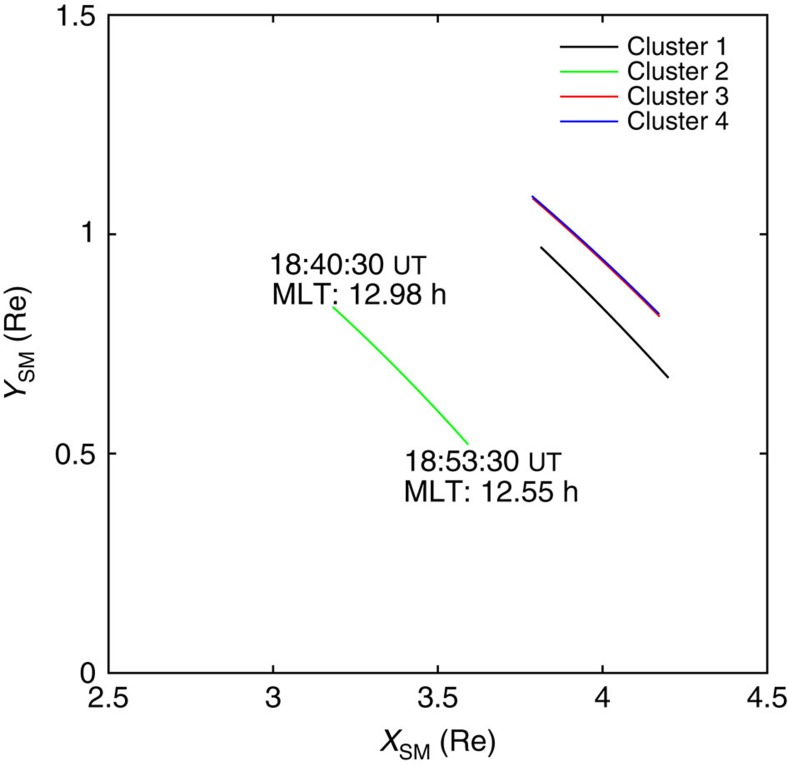
Location of Cluster spacecraft. The location and motion of the four Cluster spacecraft during the period 18:40:30 to 18:53:30 UT on 6 July 2013 in which the emissions were observed (Clusters 1, 2, 3 and 4 are shown in black, green, red and blue respectively). The coordinate system used (known as Solar Magnetic (SM)) is aligned with the Earth's magnetic field, (the *z* direction is aligned with the magnetic dipole, and the Sun direction lies in the *xz* plane) in units of Earth radii (Re). Since spacecraft Cluster 3 and Cluster 4 (C3,4) are separated by only 60 km, their traces lie virtually on top of each other. Cluster 1 is around 1,000 km from C3,4, while C2 is around 4,000 km distant.

To resolve the long-standing scientific question of the generation and propagation of the equatorial noise, ESA's Cluster mission conducted a special Inner Magnetosphere Campaign aimed at studying the structure of these waves in their source region. Figure 1 shows that on 6 July 2013, all four spacecraft were close to the geomagnetic equator. Clusters 3 and 4 were very close to each other, within 60 km, while Cluster 1 was ∼800 km from Clusters 3 and 4, and Cluster 2 was around 4,400 km in the earthward direction from the trio. Supplementary Fig. 6 and Supplementary Note 8 show the expected wavelength of the magnetosonic waves. Satellites 3 and 4 are separated by a maximum of 3–5 wavelengths, depending upon the propagation direction with respect to the separation vector.

**Figure 2:**
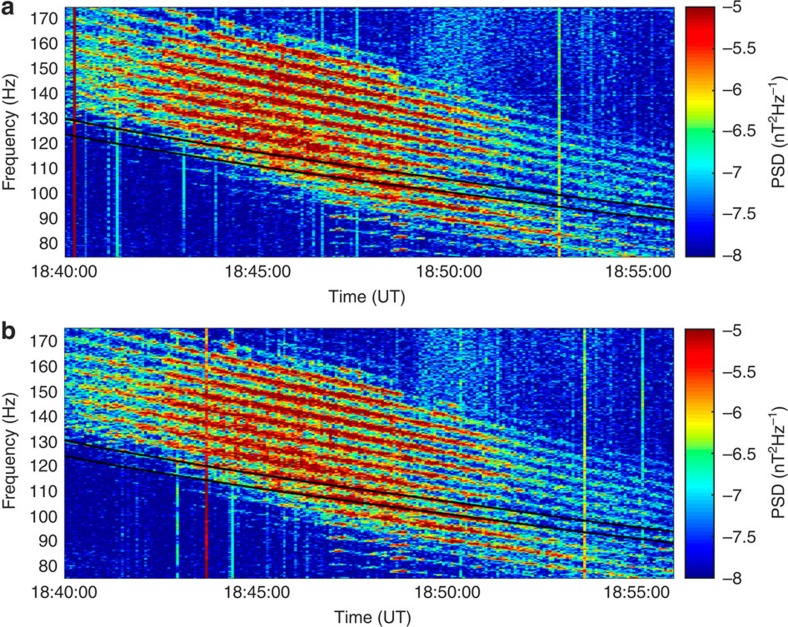
Dynamical spectrograms. Observations by the STAFF instrument on (**a**) Cluster 3 and (**b**) Cluster 4 of the harmonic structure of magnetosonic waves near the equator. The figure shows the colour coded magnetic field Power Spectral Density (PSD) as a function of time and frequency. The 20th and 21st harmonics of the proton gyrofrequency are marked by solid black lines. Harmonics up to 30th are clearly seen, and one can outline the traces of the 31st and higher harmonics.

The observations of waves made by the Cluster Spatio-Temporal Analysis of Field Fluctuations (STAFF) instrument on 6 July 2013, between 18:40 and 18:55 UT, not only present observational evidence for their generation, but also show the most remarkable example of their banded structure ever observed in space. Despite being commonly referred to as magnetosonic noise, the emissions observed by the Clusters 3 and 4 spacecraft separated by 60 km have a remarkably clear discrete structure between the 17th and 30th harmonics of the proton gyrofrequency (Fig. 2) in the frequency range in which equatorial noise is usually observed. This previously unobserved, well organized and periodic structure provides definitive evidence that these waves are generated by protons. The exact match between the harmonics and observed emissions lines shows that these observations are made right in the wave source region.

The Cluster measurements enabled not only the observation of the fine structure of the wave spectrum but also provided multi-satellite measurements of this emission at very short separation distances. The periodic pattern of emissions between the 17th and 30th harmonics observed on Cluster 4 is almost an exact replication of that observed by Cluster 3. The similarity of the signals has been analysed with the use of the coherency function (Supplementary Fig. 1 and Supplementary Note 1). The high coherence (>0.8) between the signals at harmonic frequencies of the gyrofrequency show that their separation is less than the wave coherency length and that this remarkably organized periodic structure is at least 60 km in scale which encompasses several wavelength (Supplementary Fig. 1, Supplementary Note 6).

Supplementary Fig. 2 and Supplementary Note 4 shows a comparison of the wave observations made by all four Cluster spacecrafts. While Cluster 1 observes similar discrete pattern of waves, the waves are not coherent with the Cluster 3 and Cluster 4 observations. Cluster 2 is more distant from Clusters 3 and 4, and did not observe similar type emissions.

**Figure 3:**
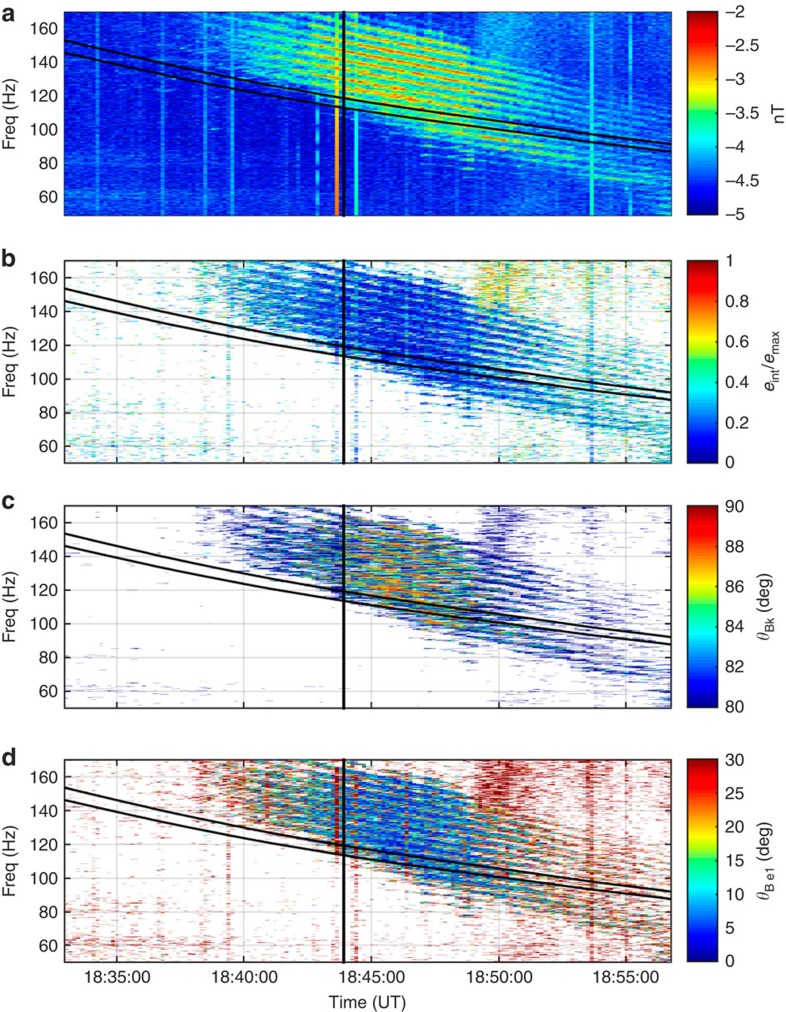
Polarization properties of the magnetosonic waves observed by Cluster 4 on 6 July 2013. (**a**) The spectrum of the waveform STAFF-SC Bz component. (**b**) The ellipticity of the waves representing the polarization of the emissions. Values close to unity indicate circular polarization while those in the region of zero are indicative of linear polarization. (**c**) The wave normal angle with respect to the external magnetic field. (**d**) The angle between the external magnetic field and the oscillating magnetic field of the wave. The horizontal black lines represent the 20th and 21st harmonics of the proton gyrofrequency. Cluster 4 crossed the geomagnetic equator at the time marked by the vertical black line.

Cluster measurements also allow to determine the polarization properties of waves to confirm that the observed emissions are the same type as the usually observed magnetosonic noise waves (Fig. 3). The fluctuating wave magnetic field on Cluster 4 is orientated parallel/antiparallel to the background magnetic field, the wave propagates at highly oblique wave normal angles, and shows linear polarization, confirming that these are typical equatorial magnetosonic waves^8^. For comparison, the polarization properties resulting from spacecrafts 1 and 3 are shown in Supplementary Figs 3 and 4.

**Figure 4:**
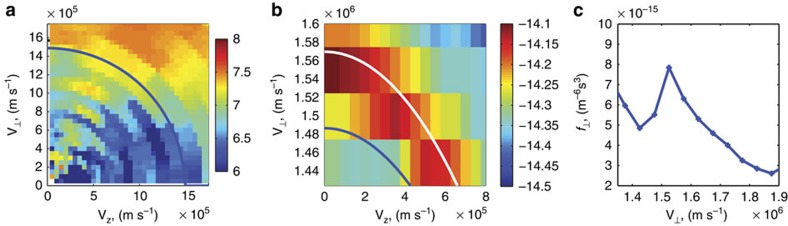
Observations of the ion distribution in velocity space. (**a**) Distributions of proton fluxes in velocity space at 18:54:33 UT. (**b**) Distribution of phase space density for quasi-perpendicular ions at 18:54:33 UT. The white line denotes velocity contour of 1.57 × 10^6^ m^−3^ while the blue line denotes a velocity contour of value equal to Alfven speed 1.487 × 10^6^ m^−3^. (**c**) A plot of the ring distribution for the phase space density of protons gyrating near the equatorial plane (particles bouncing very near the equator). The blue line connecting the data points outlines the shape of the distribution. *y* axis is the density in phase space, and *x* axis is the velocity of particles.

The Cluster spacecraft also provided an opportunity to observe the source of free energy for this wave. It has been suggested^4^,^9^ that ring-like particle distributions in velocity space may lead to wave generation through the development of instabilities. Figure 4 shows the momentum space distribution of protons near Alfvén speed (the characteristic speed at which low frequency waves propagate within a plasma) observed by the Cluster Ion Spectrometry Composition Distribution function (CIS CODIF) analyser instrument. Particle distributions at all CIS measured energies are shown in Supplementary Figs 5 and 6 and Supplementary Note 5. The observed ‘ring' distribution is unstable and results in the generation of waves^9^.

The unique observations by the multiple Cluster spacecraft in the vicinity of the geomagnetic equator clearly show the fine periodic structure of magnetosonic waves generated in their source region and the simultaneous occurrence with ‘ring-type' ion distributions.

### Excitation of waves and growth rates

The linear growth rate can be expressed as a sum of different harmonics of an integral over perpendicular velocity that depends on the gradients of ion phase space density in the velocity space, and can be expressed as^10^


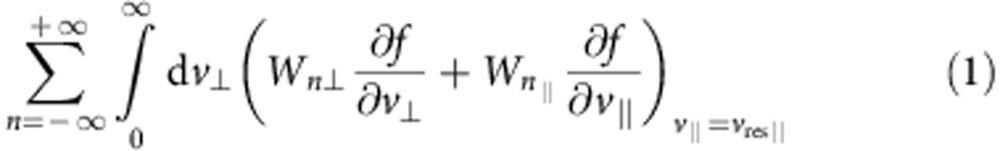


where *n* is the harmonic number, *v*_⊥_and *v*_||_ are the perpendicular and parallel velocities with respect to background magnetic field, and *W*_*n*⊥_ and *W*_*n||*_ are weighting functions. *v*_||_ in equation (1) is taken at resonance velocities corresponding to different order resonances of harmonic number *n*. Since the waves are highly oblique, the resonance occurs with the ions of *v*_||_∼0 only when the wave frequency is approximately equal to multiples of the ion gyrofrequency, while there are few resonant ions when the wave frequency is not in the vicinity of multiples of the ion gyrofrequency. The injection of protons will create a ring-type distribution, where phase space density has a positive d*f/*d*v* along the *v* direction. This ion distribution may be unstable and provide the free energy for the wave excitation with growth rate maximizing at multiples of the ion gyrofrequency.

Observations of ion distributions enable the calculation of the wave growth rates. Linear growth rates^11^ are calculated using the measured ion distributions, magnetic field and plasma density measurements inferred from wave observations. For the growth rate calculation, we use a background magnetic field *B*_0_=305 nT, electron number density of 20 × 10^6^ m^−3^ and corresponding Alfvén speed 1.487 × 10^6^ m s^−1^. To represent the observed ring, we assume a Gaussian ring distribution with number density 0.008 × 10^6^ m^−3^, peak velocity 1.57 × 10^6^ m s^−1^ and a width of 0.2 × 10^6^ m s^−1^ (as shown in Supplementary Fig. 7) and evaluate the gradient of the proton distribution in velocity space to calculate the growth rate.

**Figure 5:**
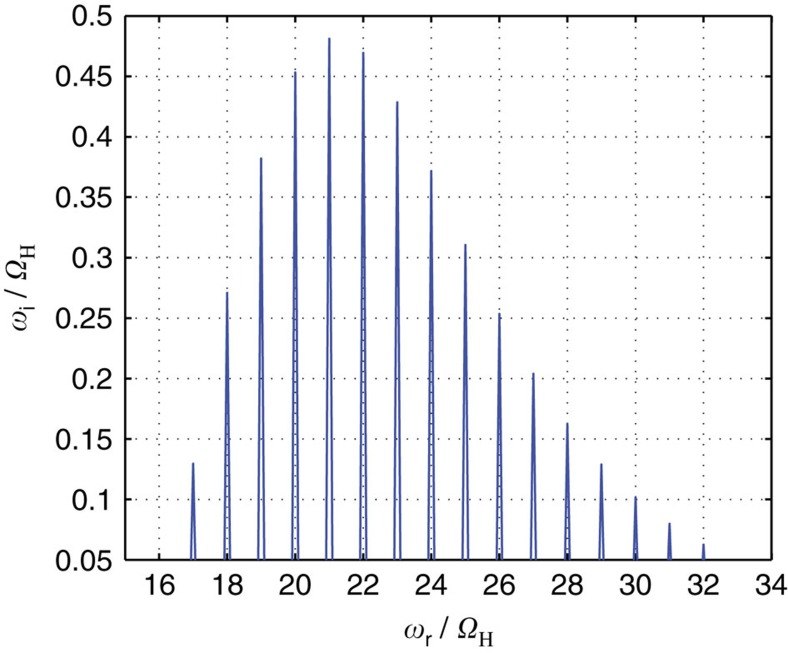
Theoretical linear growth rates based on the measured ion distributions. Growth rates *ω*_i_ normalized to the proton gyrofrequency are given as a function of wave frequency *ω*_r_ which is also normalized to the proton gyrofrequency (*Ω*_H_). Growth rate of magnetosonic waves is calculated for nearly perpendicularly directed wave vector (89.5° angle between wave vector direction and the background magnetic field).

The growth rates show the frequencies at which waves should be theoretically observed. Figure 5 shows the linear growth rate as a function of frequency normalized to the proton gyrofrequency. The general structure of peaks is very similar to those observed (as shown in Fig. 2), with maximum growth rates occurring between the 17th and 30th harmonics. Traces of the higher harmonics can be also seen in Fig. 2

## Discussion

Fast magnetosonic waves have recently attracted much attention because they are capable of accelerating particles to high energies or providing a mechanism that results in the loss of these particles into the atmosphere^5^,^12^,^13^ and may be important for space weather. The observed discrete structure of magnetosonic waves may play an important role in the acceleration and scattering of electrons and ions by these waves. The discrete nature of these waves may change how these waves interact with electrons during both gyro and bounce resonance interactions and may determine acceleration and loss rates for electrons in the radiation belts. The presence of such highly structured waves may be also used in the future as a tell-tale sign of ion ring distributions.

Similar wave generation mechanisms may also operate in the magnetospheres of the outer planets, close to the Sun and in distant corners of the universe. Understanding the mechanisms behind the generation of waves is most important for laboratory plasma and for finding new ways to remotely heat plasma.

## Methods

### Wave propagation analysis

The data sets obtained by the STAFF-SC instrument consist of a time series of vector measurements of the magnetic field. During the period of observation, the data are sampled at 450 Hz. The spectral information, shown in Fig. 2, is obtained in this particular case with the use of the fast Fourier transform technique. This results in a frequency representation of the time series data. The polarization parameters are obtained as follows. For each frequency resulting from the fast Fourier transform process, the three spectral components corresponding to the three component measurements are combined to form the spectral matrix. By analysing the complete spectral matrix using singular value decomposition (SVD) techniques^14^, it is possible to obtain the wave vector direction (without distinguishing between parallel and antiparallel directions), the orientation and the size of the polarization ellipse, and the planarity of the polarization (not shown).

SVD is a general method used to factorize a real or complex matrix, and a corresponding detailed implementation is discussed in a study^14^. This factorization process is similar to performing a least-squares fit to the data, but without actually solving the minimization problem.

### Wave growth calculation

Wave growth is calculated by solving the kinetic dispersion relation in a uniformly magnetized plasma^11^,^15^ consisting of three components: a cold electron component, a cold proton component and a hot proton ring. Several assumptions are made to facilitate the calculation using a non-Maxwellian ion distribution. First, cold plasma is dominant over the hot proton component, which allows the cold dispersion relation to be used to approximate the real part of the kinetic dispersion relation. This assumption is valid because the measured proton ring density is much smaller than the cold plasma density. Second, the growth rate is small compared with the wave frequency, which is also verified by our calculation results. With these two assumptions, we can obtain the temporal growth rate in terms of proton phase space density gradients in velocity space (as shown in the equation (1)), evaluated at resonant protons satisfying *ω*−*k*_||_ *v*_||_=*nΩ*.

### Instrumentation

The data presented in this paper were collected by the Fluxgate Magnetometer (FGM)^16^, the STAFF-SC search coil magnetometer^17^ and the CIS CODIF mass-resolving ion spectrometer^18^. During the period of observation presented here, the satellites were operating in science burst mode 1. In this mode, FGM sampled the DC magnetic field at 67 Hz whilst STAFF-SC sampled the AC magnetic field at 450 Hz through a 180 Hz filter.

## Additional information

**How to cite this article:** Balikhin, M. A. *et al.* Observations of discrete harmonics emerging from equatorial noise. *Nat. Commun.* 6:7703 doi: 10.1038/ncomms8703 (2015).

## Supplementary Material

Supplementary Information Supplementary Figures 1-8, Supplementary Notes 1-6 and Supplementary References
