# Supplementary material for: Observations of discrete harmonics emerging from equatorial noise
Source: Nat Commun. 2015 Jul 14;6:7703. doi: 10.1038/ncomms8703 (PMC4510698; doi:10.1038/ncomms8703)
Supplement: Supplementary Information — Supplementary Figures 1-8, Supplementary Notes 1-6 and Supplementary References [file ncomms8703-s1.pdf]

Supplementary Figures

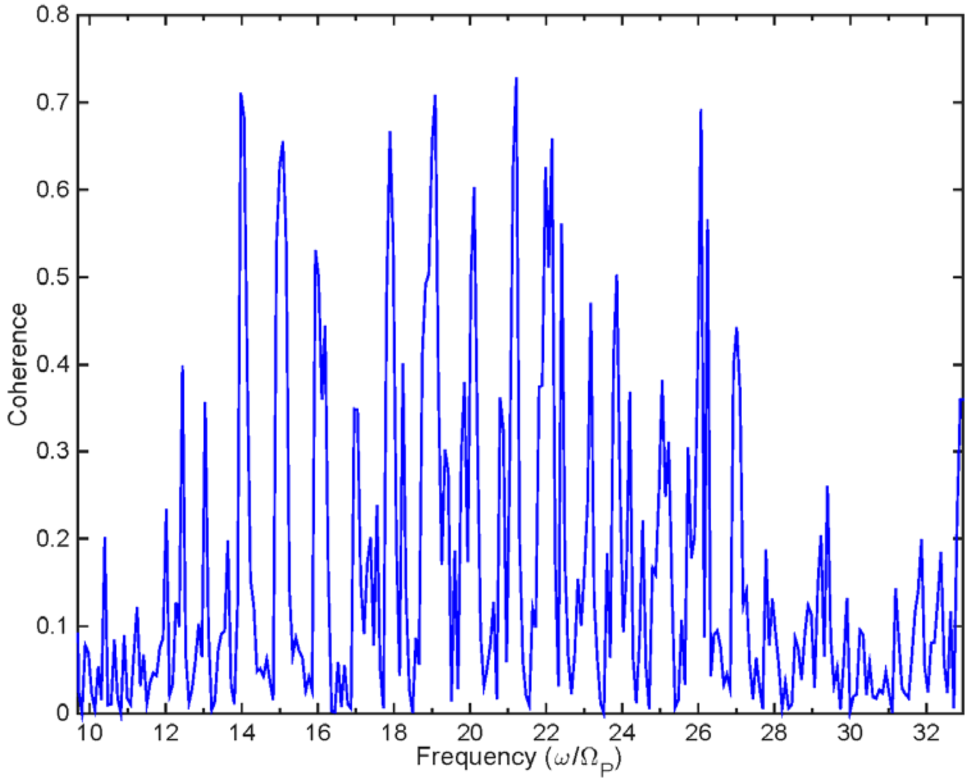

Supplementary Figure 1: Coherence between Cluster 3 and Cluster 4 measurements. Estimate of the coherence function between the B_z (SM) signals measured by C3 and C4 during the period 18:48:27 and 18:49:07 UT plotted against frequency normalized to the proton gyrofrequency.

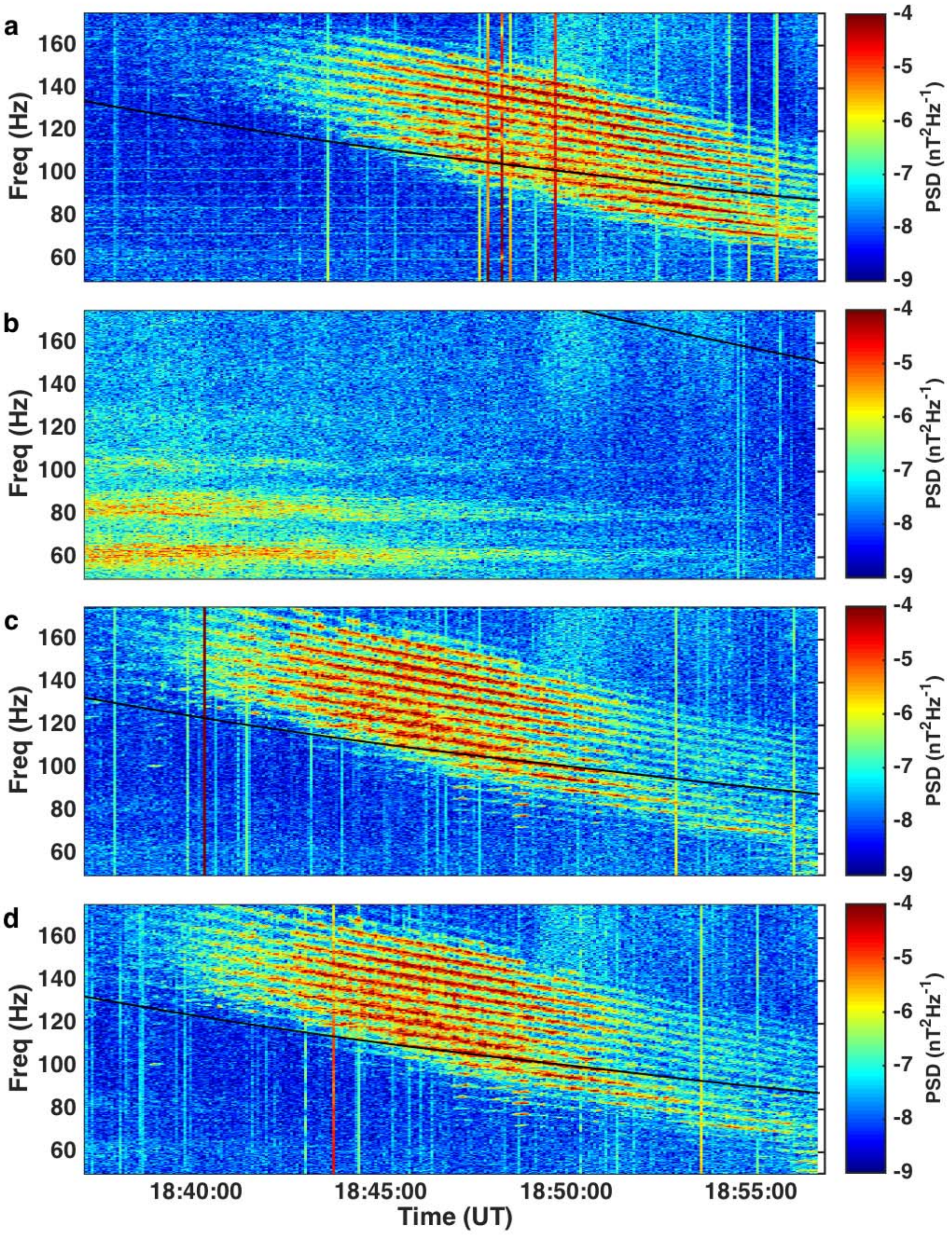

Supplementary Figure 2: The dynamical Spectra. The spectra of wave emissions observed by the STAFF instrument on Cluster (top to bottom) C1, C2, C3, and C4 on July 6, 2013.

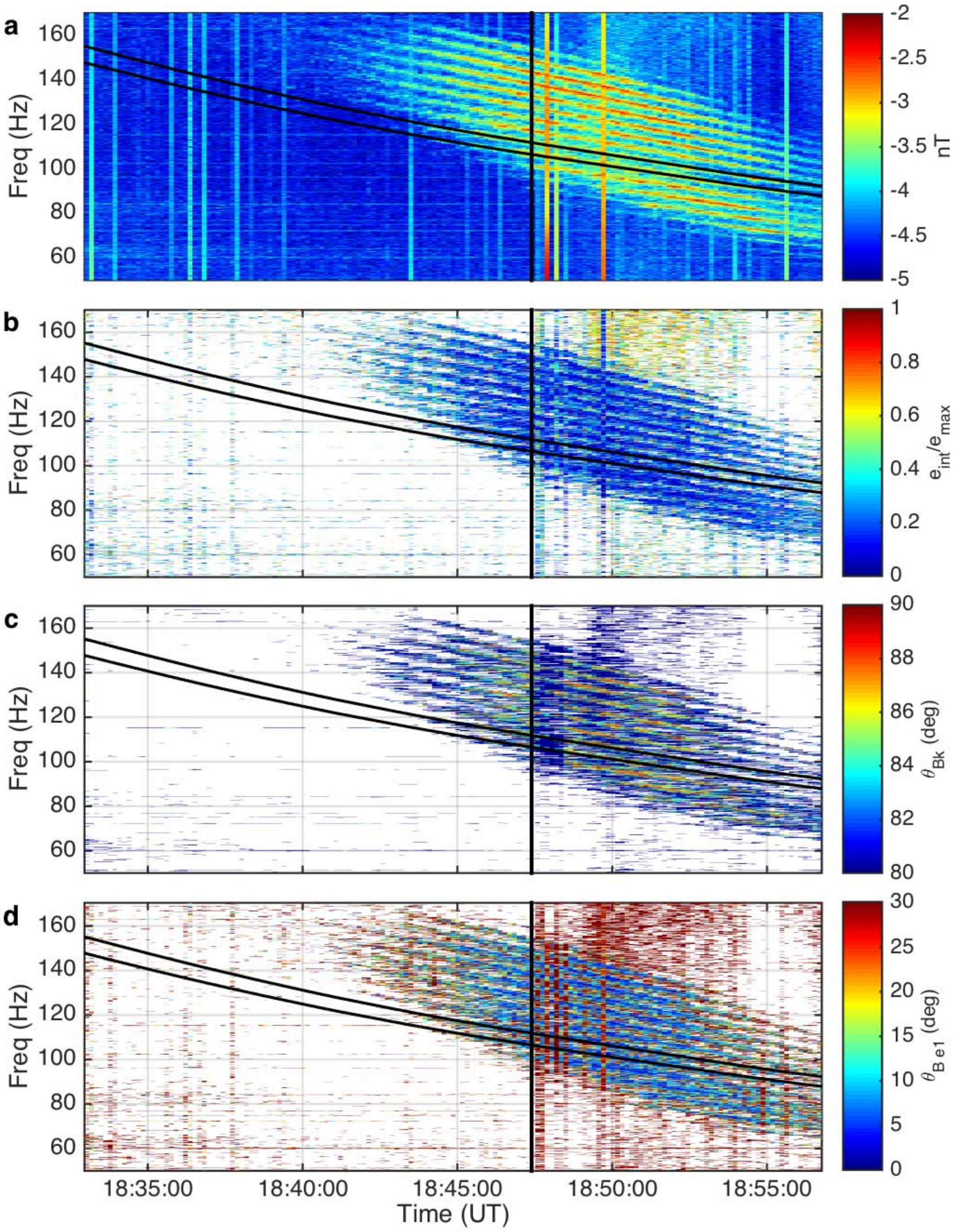

Supplementary Figure 3: **Polarization properties of the magnetosonic waves observed by Cluster 1 on July 6, 2013.** (A) the spectrum of the waveform STAFF-SC B_z component, (B) the ellipticity of the waves, (C) the wave normal angle with respect to the external magnetic field, and (D) the angle between the external magnetic field and the oscillating magnetic field of the wave. The horizontal black lines represent the 20th and 21st harmonics of the proton gyrofrequency. Cluster 1 crossed the geomagnetic equator at the time marked by the vertical black line.

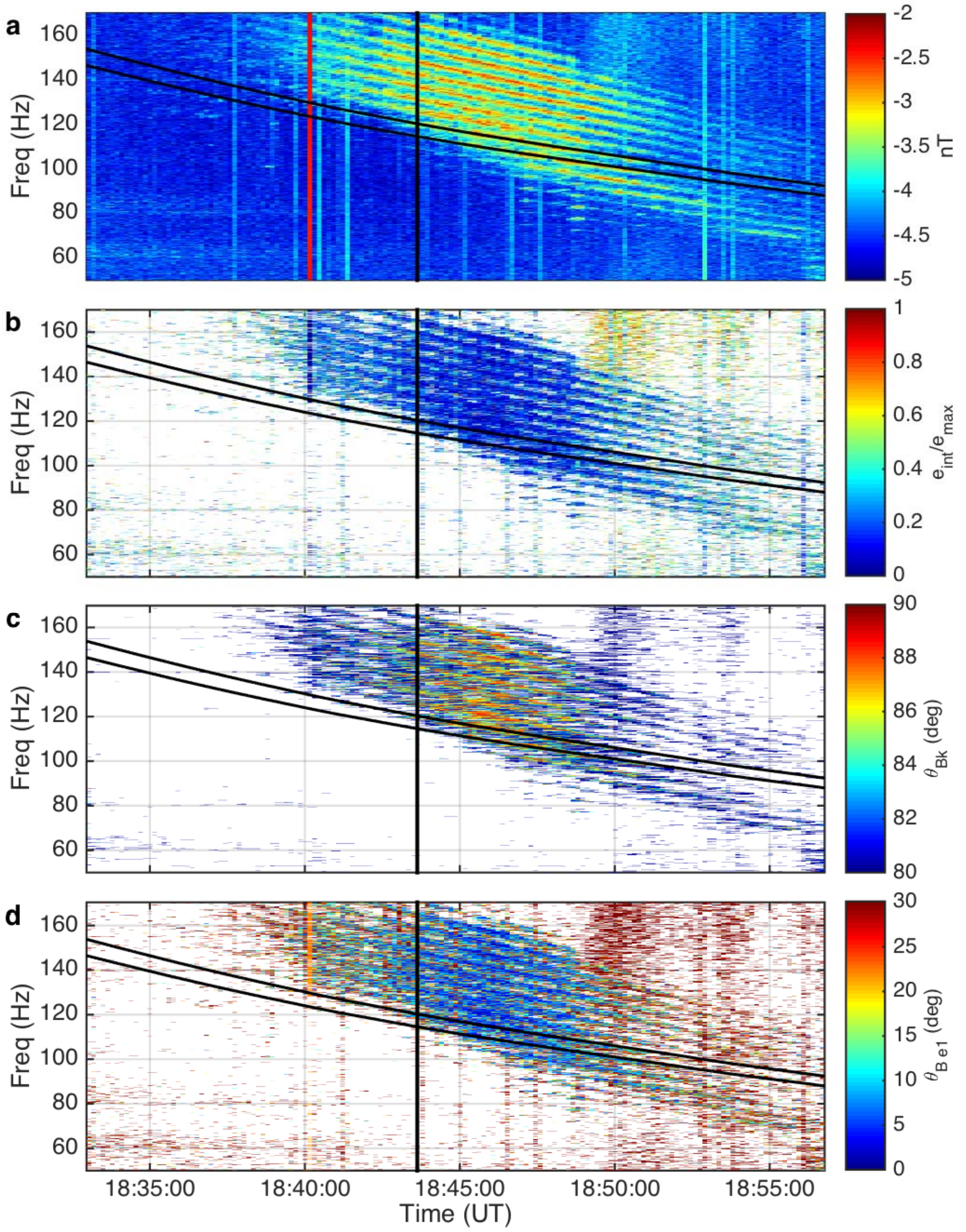

Supplementary Figure 4: Polarization properties of the magnetosonic waves observed by Cluster 3 on July 6, 2013. (A) the spectrum of the waveform STAFF-SC B_z component, (B) the ellipticity of the waves, (C) the wave normal angle with respect to the external magnetic field, and (D) the angle between the external magnetic field and the oscillating magnetic field of the wave. The horizontal black lines represent the 20th and 21st harmonics of the proton gyrofrequency. Cluster 3 crossed the geomagnetic equator at the time, which is marked by the vertical black line.

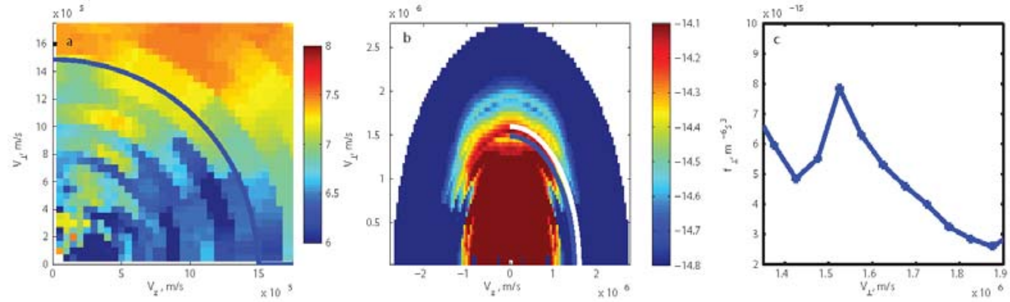

Supplementary Figure 5: Velocity space ion distributions observed simultaneously with the wave measurements. a) Distributions of proton fluxes in momentum space at 18:54:33 UT. b) Distribution of phase space density for quasi-perpendicular ions. The Alfvén speed (the characteristic speed at which low-frequency waves propagate within a plasma), is indicated by the blue line. The peaks in phase space density near the Alfvén speed were suggested to be responsible for the generation of magnetosonic waves. Such peaked distributions are usually called ‘ring’ distributions and contain free energy for the excitation of waves. The location of the ring is indicated by the white line. c) A line plot of the ring distribution for the phase space density of protons gyrating near the equatorial plane (particles bouncing very near the equator). *Y*-axis is the density in phase space, and *X*-axis is the velocity of particles.

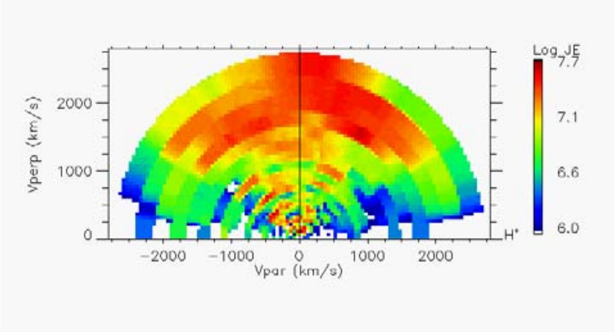

Supplementary Figure 6: Ion fluxes. Ion fluxes as observed by Cluster 4 at 18:48:30.687.

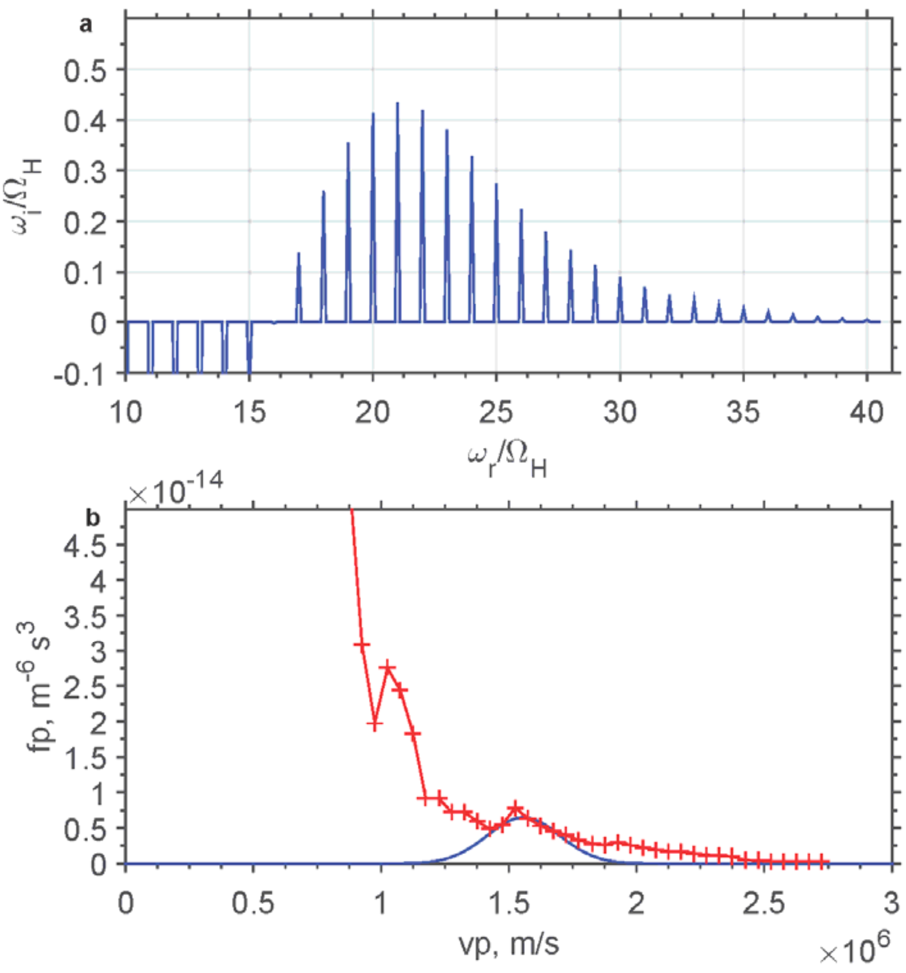

Supplementary Figure 7: Calculated growth rates. a) Results of the growth rate calculations. Significant positive growth above 0.1 is seen for resonances of 17 to 30. b) Observed distribution of the ring distribution (red line) and modeled Gaussian ring (blue line).

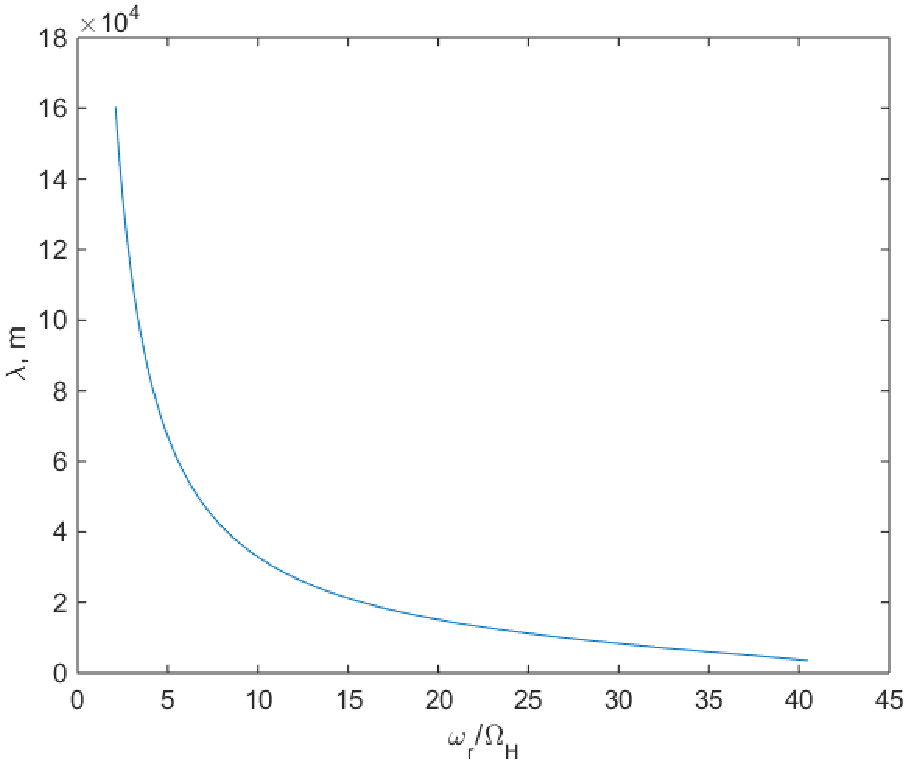

Supplementary Figure 8: Wavelength as a function of the wave frequency. Wave frequency is given in units normalized to proton gyro frequency.

Supplementary Notes

Supplementary Note 1

Supplementary Figure 1 shows the coherence function evaluated for measurements of the STAFF magnetic field (in SM coordinates) plotted as a function of frequency, normalized to the proton gyrofrequency. Since the signals of interest are constantly falling in frequency, only a short time period, 18:45:27-18:49:07, was analyzed. Supplementary Figure 1 shows that, at harmonic frequencies of the proton gyrofrequency, there is a very high coherence between the two signals measured on satellites C3 and C4 indicating that the same wave structure is observed from two vantage points. Coherence analysis is a statistical method to examine the relation between the two signals. It is defined as $C_{xy}(f) = |G_{xy}(f)|^2/G_{xx}(f)G_{yy}(f)$, where G_{xy} is the cross spectral density, and G_{xx} and G_{yy} are the auto spectral densities, resulting from the frequency decomposition of the input signals using methods such as the Fourier or Wavelet transform. A value of zero would indicate that the two signals are not related, while a value of one indicates that the signals are related by an idealized linear transfer function.

Supplementary Note 2

The first study that observed bands and structures within the magnetosonic noise was the study based on IMP6 and Hawkeye 1 data[1]. This pioneering study was the first to question the application of term noise to these emissions, and the study showed bands in the data. However,

these measurements were not taken in the source region, the lines were not very clear nor did they match the gyrofrequency, and the ring was assumed to be a delta function. The study[1] clearly stated all the above mentioned shortcomings, and in conclusion, this study described three potential explanations for the observed bands. For detailed technical difficulties associated with the analysis of the data in the study[1], please see study[2]. Another study [2] used GEOS 1 and 2 spacecraft observations. However, the instrument was only capable of observing waves up to 10 Hz, and most of the presented observations were not made exactly in the source region. Using an idealized model of a delta function ring-type distribution, the authors calculated the wave growth rates. The calculated growth rates showed clear periodic structures maximized around the 10th harmonic, which is higher than that observed by the GEOS. The wave growth calculations were calculated with an assumption of wave vector orthogonal to the magnetic field ($k_{\parallel}=0$). k denotes a wave vector, and the subscript symbol, ∥, denotes the component parallel to the magnetic field. The authors discussed a possibility that the instability is not produced by the ring but instead was produced by the loss cone distribution.

In the current study, we did not assume $k_{\parallel}=0$ and instead we used the wave normal angle of 89.5°. Cluster STAFF observations presented in this study are very clean and clear, showing high harmonics. The waves are observed exactly in their source region. There are clear emission lines (approximately 13-16 distinct and equally spaced lines), with very clear spacing between the lines that follow the gyrofrequency, leaving no doubt that we are in the source region. We use locally observed distributions of ions directly in the source region for the calculation of growth rates.

In summary, there are four major differences between the previous studies and the current study that allowed us to definitively resolve this long-standing question of the origin of these

waves: 1) We did not use a delta function distribution. Instead we use a more realistic Gaussian ring distribution to represent the observed distribution. We did not assume $k_{\parallel}=0$, 2) compared with GEOS observations, Cluster observations covered the entire frequency band, which is required to provide a definite answer to the generation mechanism. 3) The emission is in the source region because the harmonic spacing is equal to the local proton gyro frequency and the fact that changes in the emission frequency mirror the changes in the gyrofrequency. 4) Another highlight of the paper is the comparison of the growth rate based on the observed ion distribution with the observed waves over the entire frequency range and with high-frequency resolution.

A very recent study[3] searched for the harmonics of the gyrofrequency using Polar data. They analyzed the 2 kHz mode receiver data, which has a maximum resolution of 2.2 Hz. They also looked at the low-frequency wave form receiver data (0 to 25 Hz data with resolution of 0.4 Hz) and could not find the harmonic structure. One possibility is that Polar was not in the source region, and the observed waves have propagated from their source region to the point of observation, which blurs the harmonic structures.

A similar explanation has been also proposed by one study[4] which showed multipoint measurements recorded by the Cluster spacecraft mission. They observed spectral structures, which did not match the harmonics of the local proton cyclotron frequency. Analysis of polarization properties of these waves implied that their group velocity has a radial component. This observation agreed with the hypothesis of distant source regions where the cyclotron frequencies are different from the values observed at the spacecraft position.

In the current study, we have identified a very clear event in the source region where spectral structures at harmonics of the local proton cyclotron frequency are detected and the

theoretical assumptions on the origin of this important class of electromagnetic waves can be unambiguously verified.

Supplementary Note 3

The ESA Cluster mission[5] consists of four identically instrumented spacecraft. During its lifetime, the inter-satellite separation has been varied from less than a few hundred km to over 20,000 km in order to explore the processes occurring within the magnetosphere at different spatial scales.

The observations presented here were made on July 6, 2013, on the dayside of the magnetosphere at around 1330-1250 local time and a radial distance of 3.8-4.4 *Re*. The spacecraft crossed the magnetic equator at 1847 UT traveling from north to south.

Supplementary Note 4

Dynamic spectra of the observations from the STAFF-SC measurements on all four spacecraft are shown in Supplementary Figure 2. Observations on Cluster 1 also show a similarly banded periodic structure to that observed on Cluster 3 and 4. However, there are clear differences between the structure of waves since Cluster 1 enters the generation region a few minutes after Clusters 3 and 4. Observations on Cluster 2, which is over 4000 km away from the other spacecraft, show a few harmonic lines. In contrast to the Cluster 3 and Cluster 4 observations, these emissions, which are also magnetosonic in nature, occur at much lower

frequencies and also at a constant frequency rather than mirroring the changes observed in the external magnetic field at the point of observation. These frequency characteristics imply that these wave emissions have been generated at a point remote from the satellite and have propagated to the point of observation. Thus, their frequency structure is defined by the local magnetic field at the generation location. Moreover, considering the bigger separation between the harmonics, thus a larger gyrofrequency, one can infer that the source region is closer to the Earth. Going back to Cluster 1, Cluster 3 and Cluster 4 observations, the waves are mainly seen on B_x and Bz GSM components. As the main field has a very small component in the Y direction, it confirms the longitudinal polarization of the waves.

Supplementary Figures 3 and 4 show the wave polarization properties calculated based on the measurements from the Cluster 1 and 3 spacecraft. Panel (A) displays a spectrogram of the emissions. The discrete, banded nature of the emissions is clearly seen, with the bands occurring at harmonics of the proton gyrofrequency. The wave properties displayed in Panels (B), (C), and (D) of Supplementary Figures 3 and 4 have been computed using singular value decomposition (SVD) of the spectral matrix. Panel (b) shows the ratio of the intermediate to maximum eigenvalues, representing the polarization of the emissions. Values close to unity indicate circular polarization, while those in the zero region are indicative of linear polarization. Panel (b) clearly shows that this ratio is less than 0.1, evidence for the almost linear nature of emissions. Panel (c) shows the wave normal direction of the waves with respect to the background magnetic field. It clearly highlights the fact that these emissions have a wave vector in the nearly perpendicular direction, justifying the assumption of a wave normal angle used in the calculation of growth rates. Finally, panel (d) shows the angle between the maximum variance direction (representing the major axis of the polarization ellipse) and the local magnetic

field. This panel demonstrates that the wave magnetic field oscillations occur in the direction parallel to the local magnetic field. The wave properties shown in Supplementary Figures 3 and 4 are characteristic of those expected for equatorial magnetosonic noise. Analysis of the waveforms from Cluster 1 and 3 show similar results.

Supplementary Note 5

Supplementary Figure 5 shows the full distribution of the ion phase space density. The considered ring is the dominant feature near the peak Alfven speed. Other areas of high phase space density and other peaks in phase space density can be seen in Supplementary Figure 6. In the main part of the manuscript, we focused on the ring closest to the Alfven speed and high pitch angles, which are dominant for the excitation of the waves. Only ring distributions close to the Alfven speed can excite waves. In the main manuscript, we also focus on the particles close to 90° pitch angle, as there are more particles at high pitch angles than at small pitch angles.

Supplementary Figure 6 shows that the distributions were taken at 18:48:30.687. At this time, the background, due to penetrating particles from the outer radiation belt, is higher, but the ring distribution is still visible. At 18:54:33, the background was already lower, which explains the choice of the time 18:54:33 for the analysis of particle data.

Supplementary Figure 7a shows all positivity and negative growth rates, while Supplementary Figure 7b shows observations and the assumed ring for the calculations. Spectral lines below 17 are not observed, while some traces of the spectral line above 30 can be seen on dynamic spectrograms.

Supplementary Note 6

Supplementary Figure 8 shows that the wavelength is approximately 10-20 km for the given harmonic resonances. The distance between the spacecraft will be approximately 3-5 wavelengths.

Supplementary References

[1] Gurnett, D. A., Plasma wave interactions with energetic ions near the magnetic equator. *J. Geophys. Res.*, **81**, 2765–2770 (1976).

[2] Perraut, S. et al., A systematic study of ULF waves above F_H^+ from GEOS 1 and 2 measurements and their relationship with proton ring distributions. *J. Geophys. Res.*, **A87**, 6219-6236, (1982).

[3] Tsurutani, B. T. et al., Extremely intense ELF magnetosonic waves: A survey of polar observations. *J. Geophys. Res. Space Physics*, **119**, 964–977, doi:10.1002/2013JA019284 (2014).

[4] Santolik, O., Pickett, J. S., Gurnett, D. A., Maksimovic, M., & Cornilleau-Wehrlin, N., Spatiotemporal variability and propagation of equatorial noise observed by Cluster. *J. Geophys. Res.*, **107**, 1495, doi:10.1029/2001JA009159 (2002).

[5] Cornilleau-Wehrlin, N., et al., The Cluster Spatio-Temporal Analysis of Field Fluctuations (STAFF) experiment. *Space Sci. Rev.*, **79**, 107-136, 1997.
